# Implementation of Health Information Systems to Improve Patient Identification

**DOI:** 10.3390/ijerph192215236

**Published:** 2022-11-18

**Authors:** Catalin Popescu, Hani EL-Chaarani, Zouhour EL-Abiad, Iza Gigauri

**Affiliations:** 1 Department of Business Administration, Petroleum-Gas University of Ploiesti, 100680 Ploiesti, Romania; 2 Faculty of Business Administration, Beirut Arab University, Beirut P.O. Box 1150-20, Lebanon; 3 Faculty of Economic Sciences and Business Administration, Lebanese University, Beirut P.O. Box 6573/14, Lebanon; 4 School of Business, Computing and Social Sciences, Saint Andrew the First-Called Georgian University, Tbilisi 00179, Georgia

**Keywords:** healthcare, information system, information technologies, health information systems (HISs), digitalisation, patient identification, task support satisfaction, culture of patient safety

## Abstract

Wellbeing can be ensured in society through quality healthcare, a minimum of medical errors, and the improved performance of healthcare professionals. To this end, health information systems have been implemented in hospitals, with this implementation representing progress in medicine and information technologies. As a result, life expectancy has significantly increased, standards in healthcare have been raised, and public health has improved. This progress is influenced by the process of managing healthcare organizations and information systems. While hospitals tend to adapt health information systems to reduce errors related to patient misidentification, the rise in the occurrence and recording of medical errors in Lebanon resulting from failures to correctly identify patients reveals that such measures remain insufficient due to unknown factors. This research aimed to investigate the effect of health information systems (HISs) and other factors related to work-related conditions on reductions in patient misidentification and related consequences. The empirical data were collected from 109 employees in Neioumazloum Hospital in Lebanon. The results revealed a correlation between HISs and components and the effects of other factors on patient identification. These other factors included workload, nurse fatigue, a culture of patient safety, and lack of implementation of patient identification policies. This paper provides evidence from a Lebanese hospital and paves the way for further studies aiming to explore the role of information technologies in adopting HISs for work performance and patient satisfaction. Improved care for patients can help achieve health equality, enhance healthcare delivery performance and patient safety, and decrease the numbers of medical errors.

## 1. Introduction

The emergence of information technologies in healthcare offers an important means to improving services in healthcare organizations. Patient safety in healthcare is a critical issue that has yet to be adequately addressed despite continued attempts [1]. Patient safety is a division of healthcare described as the mitigation and prevention of adverse healthcare outcomes or injuries. The evolution of information technology has influenced the healthcare sector, playing a vital role in patient safety [2,3]. In the literature on patient safety, patient care information systems (PCISs) are regarded as one of the main components of a safe healthcare system [4,5].

Information technology in healthcare has been described as “the application of information processing involving both computer hardware and software for the collection, retrieval, sharing and use of healthcare information” [6]. Some of these results can be triggered by the introduction of electronic health records. One of the main objectives of electronic health records is to encourage consistency, reliability, and quality in healthcare. Additionally, these programs have demonstrated a capacity to minimize medical errors and enhance patient safety, reduce adverse drug reactions, and strengthen compliance with practice guidelines, such as through the facilitation of access to patient data, testing support, and the greater completeness and comprehensiveness of documentation [7].

In 2000, the study “To Err is Human” by the Institute of Medicine (IOM) called for the development and testing of new technologies to minimize medical errors [8], and a subsequent report from 2001, “Crossing the Quality Chasm”, called for the use of information technology as a crucial first step in improving and developing healthcare systems to achieve better and safer treatment [9]. Health information technology includes multiple tools ranging from simple charting to more sophisticated decision support and medical technology integrations. The employment of information technologies in the health sector can help increase the value of healthcare delivery, improve patient safety, reduce medical errors, and strengthen patient-to-healthcare provider engagement. A health information system is a data management system for healthcare. It includes systems that capture, store, maintain, and distribute the electronic medical records of patients, systems for the organizational management of hospitals, or decision-making systems that support healthcare policy. Thus, HISs help organizations improve data management and, hence, patients’ health while supporting decision making and the achievement of better results.

HISs make it possible to move from paper-based to computer-based data processing when using data for planning and research and to involve patients as HIS users along with healthcare professionals and hospital administrations [10,11].

In addition, health information systems also provide those systems that handle data related to providers’ and health organizations’ activities. These may be leveraged as an integrated initiative to improve patient outcomes, contribute to analyses, and influence policy making and decision making. The safety of data is a primary concern since health information systems typically access, process, or retain large volumes of sensitive data.

Health information technology involves the development of health information systems that assist in the collection, storage, and analysis of health data to help monitor the health of a population and the cost of healthcare. The data analysis enabled by the healthcare system can then improve patient care. Hospitals are aiming to achieve the same efficiencies as achieved with electronic health records on a smaller scale.

The Making Healthcare Safer Report from March 13, 2020 represents an effort by the Agency for Healthcare Research and Quality (AHRQ) to consolidate the wealth of information available in the patient safety environment into an easily accessible and actionable report [12]. The report’s goal is to offer information to clinicians, administrators, researchers, and government organizations so that they can prioritize patient safety measures. One failure can cost a hospital its reputation, destroy its presence in the market, and decrease its market share via loss of customer confidence.

Patient safety mirrors healthcare quality, including ensuring harmless service [13] through the correct identification of patients [14], as misidentification can cause harm due incorrect medical treatments or diagnoses [15]. Fortunately, the techniques and solutions available can help to reduce the risk of patient misidentification through the employment of a health information system (HIS) that standardizes patient identification approaches and spreads a culture of patient safety via its use to implement and apply policies.

HIS are often implemented to enhance the quality of care and the degree to which it is patient-centered, as well as to improve the efficiency and safety of services. The objective of health information systems is to provide improved care for patients and enhance healthcare delivery performance, increase patient safety, decrease medical errors, and strengthen patient and healthcare provider engagement.

The purpose of this study was the explore whether the health information system and other factors can improve patient safety outcomes via standardized patient identification approaches and to shed light on HISs’ advantages in providing solutions to problem reporting. The other factors include workload, nurse fatigue, a culture of patient safety, and lack of implementation of a patient identification policy. In April 2018, Neioumazloum Hospital in Tripoli, Lebanon, installed a complete set of HISs (hardware and software) in order to increase patient safety and decrease human errors related to misidentification and related consequences. A study was conducted comparing the old applied system and the HIS over two consecutive years each.

Lebanon has recently experienced the most complex crisis relating to several sectors in its entire modern history. Thus, on top of the economic and financial crisis that started in October 2019, two other major negative events were superimposed: the health crisis generated by the COVID-19 pandemic and the devastating explosion in the Port of Beirut in August 2020. Concretely, in the period 2019–2021, the nominal GDP value decreased by more than 56%. The combined impact of these critical situations had and continues to have effects in many sectors of activity, including the financial-banking sector, the health system, the social sphere, and the fields of energy, tourism and trade.

At the same time, the Lebanese medical sector has experienced quite a few challenges in the last years. First, this sector suffers from a labor shortage caused by the Lebanese financial and economic crisis that has been ongoing since 2019. The Lebanese youth working in the health sector have moved to other countries where they are better paid. This situation has created an environment of insecurity for health institutions and instability for work in the medical sector in Lebanon. Second, Lebanese employees working in the medical sector do not have a long-term career plan and are demotivated due to their high workload. Therefore, the Lebanese health sector needs to improve its managerial practices by using dynamic strategies that focus on new technologies and, especially, digitalization to solve the resource shortage.

The importance of research on the improvement possibilities offered by healthcare services has especially increased due to the COVID-19 pandemic, which also accelerated digitalization in organizations and increased demand for technological skills in employees [16,17,18]. Computer literacy and technological skills increase the readiness of healthcare professionals to use health information technologies [19].

This research contributes to enhancing patient safety, as implementing a healthcare information system can reduce misidentification and its related consequences. At the practical level, this research has significant importance for the healthcare sector, as it helps to demonstrate the benefits of HISs in helping employees to use, understand, and manage critical data collected from patients, leading to a high level of performance and, ultimately, a competitive advantage against competitors in the healthcare sector.

## 2. Literature Review

This section provides a summary of the related literature in order to explain HISs, technologies, and the implementation issues of HISs, as well as the components and advantages on which the empirical research is based.

### 2.1. Health Information Systems and Patient Safety

Patient safety is a priority in healthcare. Increasing points in the treatment cycle involves a degree of inherent uncertainty. Adverse events can be the consequence of practical problems, goods, processes, or structures. Improvements in patient safety require a comprehensive system-wide initiative involving a wide range of performance enhancement steps, environmental safety, and risk management, including infection control, safe use of medications, the safety of facilities, and safe clinical practice and a safe healthcare environment. Following the Institute of Medicine study entitled “To Err is Human: Creating a Safer Health System” [8], patient safety in healthcare organizations has gained much attention. The researchers state that if there is a safety culture in which adverse events can be reported without blaming people, they have the opportunity to learn from their mistakes and improvements can be made to prevent future human and system errors, thereby promoting patient safety [8].

Despite the widespread use of HISs, obtaining accurate, reliable, and consistent patient information remains a major challenge in healthcare [20]. The fundamental problems appear to be the lack of reliable patient information, related care programs, disease classification, and treatment planning data. While the impacts on HIS efficacy and patient medical security are clear, there are also significant implications for healthcare administration and policy making.

Erroneous patient records are caused by a variety of factors [21]. The authors have previously touched on a number of factors they consider to be among the most significant and hardest to address [22]; for example, the incompatibility of healthcare standards, inconsistent implementation of HISs, episodic HIS unavailability resulting in data duplication and inconsistency, a lack of effective data input and validation, patients spoofing or hiding information from the system for various reasons, and so on. The lack of appropriate data management practice causes medical errors [23,24].

The efficient linkage of patient health records in places of medical care and across the healthcare ecosystem to support care delivery, data interchange, analytics, and important business and clinical procedures requires accurate patient identification [25]. As health information exchange has grown over the last decade, with the healthcare industry aiming to decrease costs, improve interoperability, and shift to a patient-centric care delivery paradigm, this objective has become more important [25].

Patient-centric health systems and processes require strong information governance that ensures the integrity of patient identity and precise patient matching. Organizations should manage patient identification systems as an ongoing process, from front-end data capture to back-end quality control [25].

High-quality healthcare is impossible without patient safety, which means that patients should not suffer any health-related harm [13]. Identifying patients properly has a significant effect on patient safety, while misidentification causes critical safety issues [14].

Patient misidentification can occur in different areas, such as drug administration, surgeries, and blood transfusion [26], resulting in incorrect diagnoses, treatments, identification of pathologies, or sample collection [15].

Human and administrative errors can have effects on other factors in hospitals, including workload, nurse fatigue, the culture of patient safety, and implementation of patient identification policy. Workload influences nurses’ performance in terms of tasks and time, which in turn can affect patient safety [27,28]. Nurse fatigue is reflected in nurses’ tiredness [29]. The culture of patient safety refers to organizational norms and values that inform the direction of personnel behavior towards patients [30,31]. Lack of implementation of a patient identification policy can result in errors in patient identification and medical mistakes [32].

### 2.2. Health Information System Implementation

Researchers have used a conceptual–analytical method to explain how treatments of the significant disease ileitis might be constructed and developed into a long-term HIS architectural solution, building on earlier work and findings from the literature on use cases and case studies [33]. The authors then take this a step further by offering a healthcare architecture evaluation framework that may be used to support long-term strategic decision making; i.e., from within the evolving HIS. Reports on health information technology in recent years have shown a growing interest in health as an instrument of governance [34]. Health information infrastructures offer accurate knowledge for the implementation of policies and systems [35], using predictive indicators to forecast health outcomes and enhancing the framework of healthcare [34,36].

Health information technology can be defined as the use of information processing, including computer hardware and custody software and retrieval, classification, and use of healthcare information, records, and experience, to connect and develop solutions [37,38].

Information technology is seen as a possible perfect solution for healthcare organizations to handle the pressure to improve services in the face of increased demand. However, implementing and reviewing health information systems are riddled with challenges, and flaws and delays in implementing them are widespread [39]. HIS implementation is complex and, to be effective, must rely on organizational, financial, technical, and human factors [39]. Reflective, complex, multidimensional assessment is also needed to provide continuous input and ensure success [40].

Health information technology includes electronic health records, personal health records, clinical decision support tools, and telemedicine, as well as Internet use for data and knowledge sharing [2].

Recently, technological developments have been augmented by the provision of healthcare services on the mobile platform, enabling efficiency, accessibility and the ability to make decisions and perform diagnostic process tasks [41,42,43,44].

The delivery of healthcare services on the mobile platform is considered a positive technological development and has gained significance in the context of HISs [45,46]. With the advancement of computers and information technology, health information systems have become a significant issue [47], especially as budgetary HISs can serve the interests of physicians, patients, governments, and decision makers [48]. Furthermore, healthcare data mining can help managers make strategic decisions by allowing them to search out and predict future trends [49]. Health information technology is seen as a vehicle for improving healthcare efficiency and quality [19,50]. Health facilities are making investments in hospital information systems in the modern age of information technology, aiming to enhance efficiency of the healthcare [51,52,53].

Hospital information systems can decrease medical errors, increase efficiency and cost effectiveness, help in making prompt decisions, and improve the quality of healthcare services [54,55]. Therefore, the main objective of HISs is to reduce manual processes and improve organizational performance in terms of delivering fast and efficient health services [5,56]. Furthermore, geographic information system technology is used for epidemiological aspects of public health practice, organizing activities, and research [57].

Research on health information systems is defined as an interdisciplinary field that involves information systems, computer science, and health services [58]. On the other side, Mitchell defined health informatics as a supporting healthcare practice involving the combination of electronic and digital processes [59].

Early developments in technologies for health and medicine started with improvements in the utilities and tools being used in health services. In this context, Reichertz explained technological developments in hospitals by emphasizing the social side of technology [60]. However, it was noticed that technology must be learned, as Haux outlined [10]. Haux developed an approach that showed the importance of increasing the use and evaluation of health technologies and emphasized the need for education and research in the field of HIS [10]. Furthermore, Berg argued that success in HISs is not limited to specific criteria but depends on the implementation itself, requiring the inclusion of all parameters as systems and users [61]. In all healthcare settings, Big Data has immense potential to generate value. Organizations may use Big Data solutions to tailor care, engage patients, minimize unpredictability and costs, and improve quality [62].

Organizations can use analytics to better understand the clinical and operational conditions of their organization based on historical and current trends and anticipate what might happen in the future with a high level of dependability once Big Data are managed and connected [63].

### 2.3. Components of a Health Information System

Researchers have used a conceptual–analytical method to explain how treatments of the significant disease ileitis might be constructed and developed into a long-term HIS architectural solution, building on earlier work and findings from the literature on use cases and case studies [33]. 

HISs consist of six components [64]:

1. Resources—the legislative, regulatory, and planning frameworks required for system functionality. These include personnel, financing, logistics support, information and communications technology (ICT), and mechanisms for coordinating both within and between the six components;
2. Indicators—a complete set of indicators and relevant targets, including inputs, outputs, outcomes, determinants of health, and health status indicators;
3. Data sources—including both population-based and institution-based data sources;
4. Data management—collection and storage, QA, processing and flow, and compilation and analysis;
5. Information products—data that have been analyzed and are presented as actionable information;
6. Dissemination and use—the process of making data available to decision makers and facilitating the use of that information [64].

Most false identifications are errors arising from the suggestive police recognition procedures that are required. The healthcare sector relies on patient identification that includes the following [65]: a. name; b. assigned identification number (e.g., medical record number); c. date of birth; d. phone number; e. social security number; f. address; g. photo; h. medical records.

### 2.4. Benefits of Health Information Systems

The healthcare industry depends on a vast amount of data to make patient care decisions, facilitate service delivery, and manage the many complicated administrative tasks behind the scenes.

HIS are valuable tools that help clinicians and administrative staff ensure a seamless patient experience end-to-end. According to [66], their advantages include:

- Task support satisfaction—the degree to which the end-user believes that the information system helps in accomplishing their tasks;
- Interface satisfaction—“a measure of the human-machine interface in terms of presentation, format and information processing efficiency”;
- Compatibility—the degree to which the use of IT is perceived by a healthcare professional to be consistent with their practice style or preference;
- Collaboration—the degree to which the information system supports cross-functional or cross-organizational cooperation of end-users;
- Learnability—“the ease with which new users can begin effective interaction and achieve maximal performance”;
- Accessibility—the extent to which the user has access to the system at the time and location the user desires;
- Data analytics—HISs help gather and analyze data to manage population health and reduce healthcare costs;
- Support of collaborative care—HISs facilitate the sharing of PHI between providers and organizations, making it possible for patients to receive coordinated care from multiple providers while improving care delivery and patient outcomes;
- Cost control—By sharing information, HISs can eliminate duplicate testing and procedures, reduce time demands on staff (such as sending paper copies of patient records), and reduce costly human errors;
- Population health management—Aggregating patient data can help to identify patterns and trends, predict or prevent outbreaks, identify at-risk populations, and more;
- Clinical decision support—Integrating a patient’s individual data and medical history with broader population data and research improves diagnostics and treatment.

### 2.5. Hypothesis Development

Based on the previous work reviewed, this study investigated the importance of the adoption of HISs for patient identification and its related consequences and determined other contributing factors that may be the reasons behind misidentification.

H1.1. There is a relationship between workload and patient misidentification;

H1.2. There is a relationship between nurse fatigue and patient misidentification;

H1.3. There is a relationship between the culture of patient safety and patient misidentification;

H1.4. There is a relationship between the lack of implementation of a patient identification policy and patient misidentification;

H2.1. There is a relationship between task support satisfaction and patient misidentification;

H2.2. There is a relationship between interface satisfaction and patient misidentification;

H2.3. There is a relationship between compatibility and patient misidentification;

H2.4. There is a relationship between collaboration and patient misidentification;

H2.5. There is a relationship between learnability and patient misidentification;

H2.6. There is a relationship between accessibility and patient misidentification.

A study was undertaken in Neioumazloum Hospital in Tripoli (Lebanon) comparing the old applied system and the health information system over two consecutive years each to verify the improvement in patient identification.

## 3. Data and Research Methods

**Figure 1:**
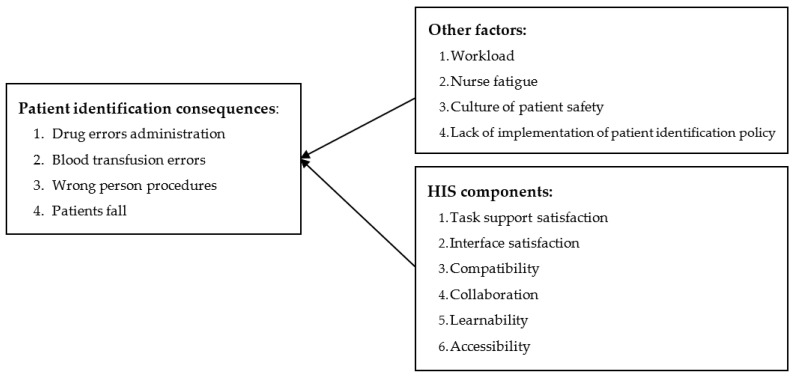
Theoretical framework of the research.

The theoretical framework was developed based on the literature review (Figure 1). It was used to investigate the relationship between the adoption of HISs and patient identification and its related consequences and to test the effects of other factors on patient identification. In this study, patient identification was treated as a unit with four factors, and there were four other separate factors: workload, nurse fatigue, culture of patient safety, and lack of implementation of a patient identification policy [67]. Furthermore, HISs were treated as including the following components: task support satisfaction, interface satisfaction, compatibility, collaboration, learnability, and accessibility.

The main purpose of this study was to test the hypotheses in order to examine the effects of applying HISs and the influence of other work-related factors on reductions in patient misidentification and related consequences. For this reason, a longitudinal study method was used, allowing a comparison between paper-based and computerized systems while testing the hypotheses. Data were collected in Neioumazloum Hospital over two consecutive years (2016–2017) to study the old-paper-based system and a further two years (2018–2019) when the computerized HIS was installed. The data were first gathered from the quality department, followed by all other departments in the hospital. Then, the data from the survey were collected to explore the relationships between various factors and patient misidentification after the introduction of the HIS in the hospital.

The first section of the questionnaire was made up of four criteria related to patient misidentification and included 16 questions in a tabular form addressed to employees to rank the gravity of misidentification consequences, with answers based on a five-point Likert scale ranging from “Always” (1) to “Never” (5).

The second section was made up of six criteria related to HIS components and included 22 questions in a tabular form addressed to employees to investigate HIS components and their impact on patient identification, with answers based on a five-point Likert scale ranging from “Does not apply” (1) to “Always applies” (5).

A quantitative study based on a survey was used in section three to study the effects of other factors contributing to patient identification. In addition, there was a section in the questionnaire for collecting demographic information related to gender, educational level, marital status, and age. The collected data were analyzed with SPSS.

### 3.1. Variables and Measures

Patient misidentification can cause different errors. Table 1 shows variables with definitions related to patient misidentification or identification errors. All the variables were measured using a Likert scale ranging from 1 to 5.

Table 2 shows variables measuring the other factors that may influence healthcare services provided to patients, along with the conceptual and operational definitions.

Table 3 defines variables related to HIS components that influence patient identification, along with their conceptual and operational definitions.

### 3.2. Sample Description

A total of 109 employees from Neioumazloum Hospital answered the questionnaire, providing data concerning the other factors contributing to misidentification and their opinions regarding HIS benefits. A total of 34 employees of the hospital were excluded from this study due to their not having direct involvement in the medical and information system. The number of employees in the hospital is 143. Therefore, 76.2% of employees in the hospital were involved in this research.

In addition, more than 200 data points from incident and accident sheets concerning misidentification were collected from the quality department in Neioumazloum Hospital for the period extending from 2016 to 2019. The data from the incident and accident sheets were provided to us without the names of patients and after approval was obtained from the administration. The survey included the informed consent of participants and was ethically approved by the staff and management of the hospital. The personnel had the freedom to express their opinions independently from the management of the hospital.

## 4. Research Results

Data analysis was performed in SPSS, including descriptive statistics, a reliability test using Cronbach’s alpha, linear multiple regressions, and correlation.

The descriptive statistics test was conducted to determine the frequency distribution of the respondents in term of the demographic factors gender, age, marital status, and educational level (Table 4).

Of the 109 employees who completed the questionnaire, the majority of respondents were female (76.1%), and they were mostly aged between 20 and 39 (33.94–42.2%). Additional, 58.72% were single, 47.71% held a bachelor’s degree, and 55.56% had a work schedule of 12 h. Twenty-two questions examined HIS components, the responses of which ranged between the rankings 2 (“Disagree”) and 5 (“Strongly agree”) (Table 5). The means of the majority of factors did not reach 4 (“Agree”).

Twenty-two questions were asked to evaluate the effects of other factors (workload, nurses’ fatigue, culture of patient safety, and lack of implementation of patient identification policy) from the employee perspective (Table 6). The results showed that the answers were between the rankings 2 (“Rarely”) and 5 (“Always”). The mean values for all factors were near to the ranking 4 (“Most of the time”).

Cronbach’s alpha is the most common measure of internal consistency (“reliability”) among items on a scale. Reliability includes the stability and consistency of measures. The reliability of a measure indicates the extent to which the measure is free of error. The Cronbach’s alpha was thus calculated for each item (Table 7).

According to [71], the Cronbach’s alpha coefficient should be greater than 0.5 in order to be acceptable. As shown in Table 8, the Cronbach’s alpha values were 0.812 and above, demonstrating that the reliability among items was consistent.

The Cronbach’s alpha values shown in Table 8 were 0.682 and above, indicating that the reliability among items was consistent.

The *p*-value for the correlation between HIS components and other factors’ effects was 0.012, demonstrating strong evidence that the HIS components were linearly correlated with the evaluation of the other factors. The significant Pearson correlation coefficient value of 0.307 indicated a strong positive correlation between the two variables (Table 9).

Multiple regression analysis was used to test the hypotheses and determine the relationship between the dependent variable and multiple independent variables. The general equation is the following:

Y = a + b_1_ × X_1_ + b_2_ × X_2_ + b_3_ × X_3_ + b_4_ × X_4_ + b_5_ × X_5_ (1)

where:

Y is the dependent variable;

a is the constant—it is the amount of Y when X = 0;

b_n_ is the slope, which can be positive or negative;

X_n_ is the dependent variable.

Coefficients showing a higher beta are the most influential variables in a model. The results showed that nurse fatigue was the most important independent variable with a higher beta of 0.451, while lack of implementation of patient identification policy was the second most important variable influencing misidentification with a beta of 0.239 (Table 10).

The third factor was the culture of patient safety with a beta = 0.093, indicating less importance than the second factor.

The fourth factor, workload, with a beta of −0.438, was the least important in the equation, which means that for every one-unit increase in the workload variable, the misidentification variable decreased by the beta coefficient value. Comparing the significance levels of the four variables to 5% showed that all variables had a significance level lower than 5%.

Misidentification = 2.304 − 0.438 (workload) + 0.451 (nurse fatigue) + 0.093 (culture of patient safety) + 0.239 (lack of implementation of patient identification policy) + e (error).

As shown in Table 11, the R value = 0.348, the R squared = 0.121, and the adjusted R squared = 0.069.

R squared shows that 12.1% of misidentification could be explained by HIS components. This means that 87.9% of the variation in the dependent variable was explained by other variables.

Since F = 2.339 and the significance value was 0.037 (*p* < 0.05), the model as a whole was statistically significant (Table 12).

The results show that task support satisfaction was the most important independent variable, having a higher beta of 0.884, while learnability was the second most important variable influencing misidentification, with a beta of 0.403 (Table 13). The third factor was accessibility, with a beta of 0.224, indicating less importance than the second factor. The fourth factor, compatibility, had a beta of 0.080 and did not have a very strong influence on misidentification.

The last two factors, interface satisfaction and collaboration, had the least importance in the equation, with beta values of −0.800 and −0.504, meaning that, for every one-unit increase in the interface satisfaction and collaboration variables, the misidentification outcome decreased by the beta coefficient value. Comparing the significance levels of the five variables to 5% showed that all variables had a significance level lower than 5%.

The following model was derived from the analysis of coefficients:

Misidentification = 2.456 + 0.884 (task support satisfaction) − 0.800 (interface satisfaction) + 0.080 (compatibility) − 0.504 (collaboration) + 0.403 (learnability) + 0.224 (accessibility) + e (error).

Four regression coefficients were positive and statistically significant.

**Figure 2:**
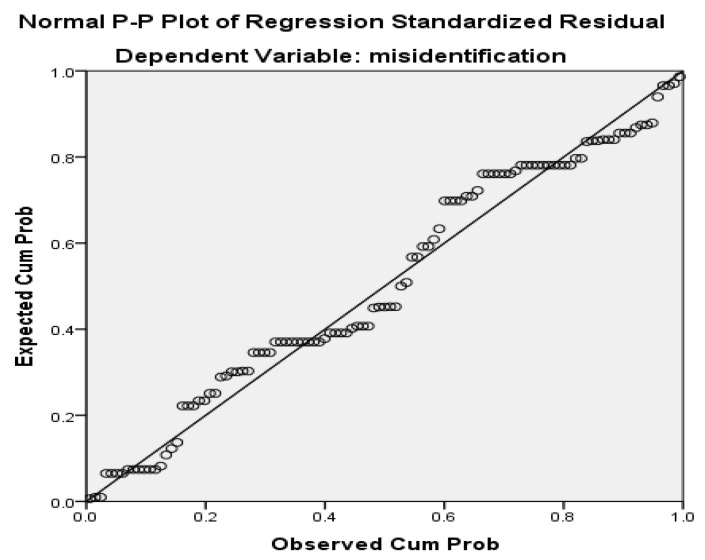
Normal P = plot of regression-standardized residual.

According to [72], a plot is a graphical technique to assess whether data are normally distributed. Figure 2 presents a plot showing the cumulative distribution of the actual values of X against the theoretical values of X under the normal distribution. The data plotted form an approximate straight line and show a positively skewed distribution.

## 5. Discussion 

HISs have become a crucial part of medical and health research as the advancement of digital technology has extended HIS studies. Healthcare reforms are focused on improving healthcare services and patient satisfaction through enhanced management processes and updated technologies [73]. Healthcare specialists need to process medical data and use technologies to avoid errors and for assistance in decision making [23]. HIS technologies offer improved opportunities, such as decreasing human and medical errors, improving performances and results, tracking data, coordinating health services, and achieving better clinical outcomes [2,74]. In addition, healthcare information systems enable timely, quality, and efficient service [23,75]. Moreover, human capital affects organizational performance [76,77,78].

This research examined the effects of the adoption of HIS and other factors on patient identification in Neioumazloum Hospital. The data collected through the survey for this study included the years 2018–2019, after an HIS was introduced in the hospital. The implementation of an HIS is an important change in the operating environment of a hospital and, hence, the measurement of the potentially related factors covered the period after the introduction of the HIS. In testing the relationship between patient misidentification and both the HIS adoption and the other factor components, the following results were obtained (Table 14).

Thus, the research provides evidence of relationships between various factors and patient identification that need to be addressed to reduce errors related to patient identification and its related consequences that might be severe and cost patients their lives, such as by reducing nurse fatigue, increasing the culture of patient safety, and implementation of a clear policy for patient identification. Furthermore, it provides evidence of a relationship between HIS components and patient identification that must be fortified with frequent and continuous training sessions and follow-up for all concerned to stress that they must go deep beyond ignoring errors to reach data analysis that enhances their knowledge, performance, and work. As the results demonstrate, addressing the other factors can increase the benefits of HIS adoption and reduce errors related to patient identification.

The findings of this research resonate with prior studies explaining the relationship between HISs and healthcare professionals [62,75,79,80] and patient misidentification factors [24,54,81].

## 6. Conclusions 

The healthcare sector is a difficult and delicate place to work in that involves dealing with customers, patients’ suppliers, colleagues, guarantors, and the latest developments, in addition to the daily problems and difficulties that need special treatment, and as all of these seem to be big challenges, it is sometimes hard to keep a positive attitude,.

A patient identification infrastructure must be viable in the long term, and there is limited research on successful long-term IT infrastructure building with significant social and political factors influencing its success. Today’s technologies will change at a rapid pace; therefore, a long-term solution must include the system’s ability to evolve without causing unacceptable disruption.

The proposed framework is a method that may be used to evaluate ideas for the enhancement of patient identification in terms of resolving the most important stakeholder issues, which are not often expressed directly as non-functional requirements. Government agencies (e.g., health departments, health district management), NGOs (e.g., the WHO, standards bodies, etc.), and even HIS vendors may be end-consumers of this framework.

Based on the results obtained, management in Neioumazloum Hospital should take into consideration the following recommendations in order to reach high performance in terms of patient identification:

- Health professionals need to be educated with a critical perspective on what the HIS can do for them. People tend to project “intelligence” and “objectivity” onto computers, and physicians and nurses are no exception. Thus, all employees and even doctors need continuous training in HIS modules in order to be up-to-date on all new information and new modules and manage their work correctly;
- Management and HR should put more effort into attracting and hiring more professional nurses with computer skills to reduce the negative consequences of the other factors;
- The representative continuous assistance and presence of an HIS are mandatory in order to align all needed information among current and new employees;
- Management and HR should review employees’ working hours in order to reduce nurse fatigue factors.

This research can be also useful for other hospitals in developing countries. Given that the adoption of HISs in developing countries is at the initial stage [5,82,83], this research can contribute to their development in practice and theory. The findings could be beneficial for hospitals considering the adoption of HISs that are making efforts to improve the quality of their services while reducing errors. The implementation of the HIS revolutionized the operating contexts of the Neioumazloum Hospital and the healthcare sector in Lebanon by providing a platform for better service, as its use reduces errors in hospitals.

Moreover, healthcare managers can make decisions regarding HISs based on this research. As the adoption of HIS requires government support, financial resources, the enthusiasm of top management, and acceptance of doctors and nurses [81,84,85,86,87,88,89], this research offers additional incentives in favor of the introduction of HISs in hospitals. The findings of this study should be advantageous for healthcare practitioners, decision makers, managers, and service providers. Hospitals’ management and administrators can improve the efficiency and effectiveness of their services using HISs. Decision makers can develop appropriate policies and strategies to facilitate the adoption of HISs and proper infrastructure for the advancement of technologies in healthcare. In addition, system developers can also benefit from this study and improve programs.

The main limitation of this research is that it was based on a case study approach and qualitative survey that investigated 109 employees in only one hospital in Lebanon. Further studies should base work on this research and examine the contexts in other countries, including with more hospitals and healthcare professionals. In addition, the relationship between HIS components and other factor components considered in this research could also include demographic factors affecting the successful implementation of HISs. Moreover, further studies could comprehensively explore the extent to which factors such as workload and nurse fatigue apply to healthcare professionals in relation to their positions and roles in healthcare organisations. Finally, the process of the introduction of HISs into hospitals could be extensively investigated through qualitative and quantitative methods to reveal the whole process and the changes it brings about.

## Figures and Tables

**Table 1:** Consequences of patient misidentification with their conceptual and operational definitions.

Variable	Conceptual Definition	Used Items
Drug administration errors	“a failure in the treatment process that leads to, or has the potential to lead to, harm to the patient” [68,69]	4
Blood transfusion errors	The transfer of blood or blood components from one person (the donor) into the bloodstream of another person (the recipient)	4
Procedures with the wrong person	Wrong surgery, wrong results, a wrong diagnosis that encompasses a medical act performed on the wrong patient or wrong side	4
Patient falls	“A fall is defined… as a sudden unexpected descent from a standing, sitting, or horizontal position, including slipping from a chair to the floor, a patient found on the floor, and an assisted fall” [70]	3

**Table 2:** Other factors affecting healthcare service.

Variable	Conceptual Definition	Used Items
Workload	Nursing workload affects the time that a nurse can allot to various tasks. Under a heavy workload, nurses may not have sufficient time to perform tasks, which can have a direct effect on patient safety [27,28]	5
Nurse fatigue	Fatigue is defined as “physical and/or mental exhaustion that can be triggered by stress, medication, overwork or mental and physical illness or disease” [29]	5
Culture of patient safety	A culture of patient safety is “a component of organizational culture, includes the shared beliefs, attitudes, values, norms and behavioural characteristics of employees, which influences staff member attitudes and behaviours in relation to their organization’s ongoing patient safety performance” [30,31]	4
Lack of implementation of patient identification policy	“An approach to avoiding patient misidentification for the prevention of medical errors, which include errors in medication, transfusion, and testing, as well as wrong-person procedures and the discharge of infants to the wrong family” [32]	8

**Table 3:** HIS components.

Variable	Conceptual Definition	Used Items
Task support satisfaction	The degree to which the end-user believes that the information system helps in accomplishing their tasks [66]	5
Interface satisfaction	“[A] measure of the human-machine interface in terms of presentation, format and information processing efficiency” [66]	4
Compatibility	The degree to which the use of IT is perceived by a healthcare professional to be consistent with their practice style or preference [66]	4
Collaboration	The degree to which the information system supports cross-functional or cross-organizational cooperation of end-users [66]	3
Learnability	“[T]he ease with which new users can begin effective interaction and achieve maximal performance” [66]	3
Accessibility	The extent to which the user has access to the system at the time and location the user desires [66]	3

**Table 4:** Demographic factor analysis for respondents.

Demographic		Frequency	Percentage	Cumulative Percentage
Gender	Female	83	76.1	100.0
	Male	26	23.9	23.9
Age	20–29	37	33.94	33.94
	30–39	46	42.20	76.15
	40–49	21	19.27	95.41
	50 or over	5	4.59	100.00
Marital status	Single	64	58.72	58.72
	Married	45	41.28	100.00
Educational level	Less than high school	1	0.92	33.94
	High school diploma or equivalent	21	19.27	76.15
	Bachelor’s degree	52	47.71	95.41
	Master’s degree	35	32.11	100.00
Schedule	8 hours	49	44.44	44.44
	12 hours	60	55.56	100.00
Department	ER	9	7.32	18.70
	Medical imaging	5	4.07	22.76
	Administrative	8	6.50	29.27
	External clinics	2	1.63	30.89
	Cath lab	4	3.25	34.15
	Surgery	16	13.01	47.15
	Pre-op	13	10.57	57.72
	OR	5	4.07	61.79
	MED I	10	8.13	69.92
	MED II	10	8.13	78.05
	Cardio	13	10.57	88.62
	Lab	14	11.38	100.00

**Table 5:** HIS component descriptive statistics.

	N	Range	Minimum	Maximum	Mean	Std. Deviation	Variance
Task support satisfaction	109	2.0	3.0	5.0	3.936	0.2814	0.079
Interface satisfaction	109	2.0	3.0	5.0	3.853	0.3807	0.145
Compatibility	109	2.0	3.0	5.0	3.771	0.4437	0.197
Collaboration	109	2.0	3.0	5.0	3.651	0.4977	0.248
Learnability	109	2.0	3.0	5.0	3.761	0.4493	0.202
Accessibility	109	2.0	3.0	5.0	3.853	0.3807	0.145
Valid N (listwise)	109						

**Table 6:** Descriptive analysis for other factors.

	N	Range	Minimum	Maximum	Mean	Std. Deviation	Variance
Workload	109	2.0	3.0	5.0	3.844	0.3891	0.151
Nurse fatigue	109	2.0	3.0	5.0	3.358	0.5004	0.250
Culture of patient safety	109	3.0	2.0	5.0	3.688	0.5392	0.291
Lack of implementation of patient identification policy	109	2.0	3.0	5.0	3.706	0.4773	0.228
Valid N (listwise)	109						

**Table 7:** Cronbach’s alpha coefficients for HIS component variables.

Item-Total Statistics		
	Number of Items	Cronbach’s Alpha if Item Deleted
Task support satisfaction	5	0.900
Interface satisfaction	4	0.844
Compatibility	4	0.846
Collaboration	3	0.835
Learnability	3	0.812
Accessibility	3	0.860

**Table 8:** Cronbach’s alpha coefficients for other factors affecting healthcare service.

Item-Total Statistics		
	Number of Items	Cronbach’s Alpha if Item Deleted
Workload	5	0.711
Nurse fatigue	5	0.876
Culture of patient safety	4	0.682
Lack of implementation of patient identification policy	8	0.688

**Table 9:** Correlations between HIS components and other factors’ effects.

		Other Factors’ Effects
HIS components	Pearson Correlation	0.307 **
	Sig. (two-tailed)	0.012
	N	109

**, Correlation was significant at the 0.01 level (two-tailed).

**Table 10:** Coefficients.

Model		Unstandardized Coefficients		Standardized Coefficients	t	Sig.	95.0% Confidence Interval for B	
		B	Std. Error	Beta			Lower Bound	Upper Bound
1	Constant	2.304	0.443		5.201	0.000	1.425	3.182
	Workload	−0.438	0.088	−0.453	−4.957	0.000	−0.613	−0.263
	Nurse fatigue	0.451	0.153	0.435	2.942	0.004	0.147	0.754
	Culture of patient safety	0.093	0.134	0.101	0.694	0.009	−0.173	0.359
	Lack of implementation of patient identification policy	0.239	0.093	0.222	2.580	0.011	0.055	0.422

**Table 11:** Multiple regression: patient misidentification consequences and HIS components.

Model	R	R Squared	Adjusted R Squared	Std. Error of the Estimate
1	0.348 ^a^	0.121	0.069	0.51802

^a^. Predictors: constant, accessibility, compatibility, interface satisfaction, collaboration, learnability, task support satisfaction.

**Table 12:** ANOVA misidentification and HIS components.

Model		Sum of Squares	df	Mean Square	F	Sig.
1	Regression	3.766	6	0.628	2.339	0.037
	Residual	27.372	102	0.268		
	Total	31.138	108			

**Table 13:** Coefficients for the HIS.

Model		Unstandardized Coefficients		Standardized Coefficients	t	Sig.	95.0% Confidence Interval for B	
		B	Std. Error	Beta			Lower Bound	Upper Bound
1	Constant	2.456	0.540		4.545	0.000	1.384	3.527
	Task support satisfaction	0.884	0.604	0.627	1.462	0.007	−0.315	2.082
	Interface satisfaction	−0.800	0.567	−0.580	−1.410	0.002	−1.926	0.326
	Compatibility	0.080	0.121	0.075	0.562	0.009	−0.161	0.321
	Collaboration	−0.504	0.200	−0.506	−2.517	0.013	−0.902	−0.107
	Learnability	0.403	0.250	0.358	1.615	0.001	−0.092	0.898
	Accessibility	0.224	0.384	0.187	0.483	0.004	−0.538	0.986

**Table 14:** Hypotheses verification.

Hypothesis Number	Hypothesis Description	Result
H1.1.	There is a relationship between workload and patient misidentification	Not accepted
H1.2.	There is a relationship between nurse fatigue and patient misidentification	Accepted
H1.3.	There is a relationship between the culture of patient safety and patient misidentification	Accepted
H1.4.	There is a relationship between the lack of implementation of patient identification policy and patient misidentification	Accepted
H2.1.	There is a relationship between task support satisfaction and patient misidentification	Accepted
H2.2.	There is a relationship between interface satisfaction and patient misidentification	Not accepted
H2.3.	There is a relationship between compatibility and patient misidentification	Accepted
H2.4.	There is a relationship between collaboration and patient misidentification	Not accepted
H2.5.	There is a relationship between learnability and patient misidentification	Accepted
H2.6.	There is a relationship between accessibility and patient misidentification	Accepted

## Data Availability

Not applicable.
